# Microbial Life in a Fjord: Metagenomic Analysis of a Microbial Mat in Chilean Patagonia

**DOI:** 10.1371/journal.pone.0071952

**Published:** 2013-08-28

**Authors:** Juan A. Ugalde, Maria J. Gallardo, Camila Belmar, Práxedes Muñoz, Nathaly Ruiz-Tagle, Sandra Ferrada-Fuentes, Carola Espinoza, Eric E. Allen, Victor A. Gallardo

**Affiliations:** 1 Marine Biology Research Division, Scripps Institution of Oceanography, University of California San Diego, La Jolla, California, United States of America; 2 Center for Optics and Photonics, Universidad de Concepción, Concepción, Chile; 3 Programa de Magister en Ciencias del Mar, Facultad de Ciencias del Mar, Universidad Católica del Norte, Coquimbo, Chile; 4 Departamento de Biología Marina, Facultad de Ciencias del Mar, Universidad Católica del Norte, Coquimbo, Chile; 5 Centro de Estudios Avanzados en Zonas Aridas, La Serena, Chile; 6 Centro de Biotecnología, Universidad de Concepción, Concepcion, Chile; 7 Laboratorio de Genética y Acuicultura, Departamento de Oceanografía, Universidad de Concepción, Concepcion, Chile; 8 Laboratorio de Bentos, Departamento de Oceanografía, Universidad de Concepción, Concepcion, Chile; 9 Division of Biological Sciences, University of California San Diego, La Jolla, California, United States of America; Universidad Miguel Hernandez, Spain

## Abstract

The current study describes the taxonomic and functional composition of metagenomic sequences obtained from a filamentous microbial mat isolated from the Comau fjord, located in the northernmost part of the Chilean Patagonia. The taxonomic composition of the microbial community showed a high proportion of members of the *Gammaproteobacteria*, including a high number of sequences that were recruited to the genomes of *Moritella marina* MP-1 and *Colwellia* *psycherythraea* 34H, suggesting the presence of populations related to these two psychrophilic bacterial species. Functional analysis of the community indicated a high proportion of genes coding for the transport and metabolism of amino acids, as well as in energy production. Among the energy production functions, we found protein-coding genes for sulfate and nitrate reduction, both processes associated with *Gammaproteobacteria*-related sequences. This report provides the first examination of the taxonomic composition and genetic diversity associated with these conspicuous microbial mat communities and provides a framework for future microbial studies in the Comau fjord.

## Introduction

The Comau fjord is located in the northernmost part of the austral region of Chile, approximately 80 km south of the city of Puerto Montt. With a length of about 45 km, a width of about 5 km and a north–south orientation, the Comau fjord is comparatively smaller than others fjords in the country, but is one of the deepest (~490 m). The surrounding hills are covered by a cold-temperate rain forest that reaches up to 2,000 m in elevation. The high precipitation rate (~6,000 mm a year) provides an input of fresh water resulting in a surface layer with estuarine properties subject to seasonal variations in depth (up to 10 m during the rainy season), with temperatures ranging between 8–12 ^°^C [1]. In addition, this input of fresh water provides minerals, metals and organic compounds to the aquatic ecosystem, captured during its passage through the ground and rocks from the surrounding hills [1,2]. The region that delimits the Comau fjord has a history of volcanic activity, manifested in the presence of large number of volcanoes, geysers and thermal springs, all of which also provide an input of nutrients and inorganic compounds into the system [3].

**Figure 1:**
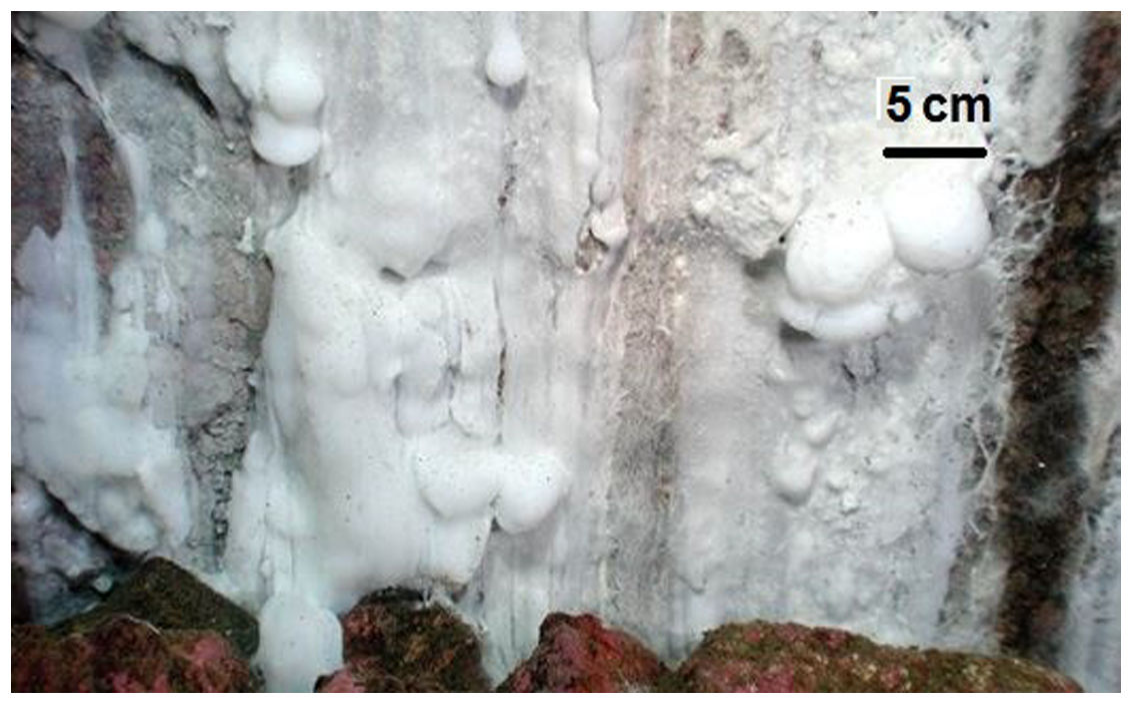
Patches of white cotton-like microbial mats on the rocky walls of the Comau fjord. (Photo by Verena Häussermann and Günter Försterra).

In 2003, patches of large filamentous bacteria forming white cotton-like microbial mats were discovered at shallow depths, between 20 to 30 m, attached to the rocky walls of the Comau fjord [4] (Figure **1**). Water composition analysis, shows that low temperature, sulfide-rich fluid seeps from the rocks with chemical compositions that resembling that of cold vents, and this may be the source of chemical energy sustaining these microbial formations (Javier Sellanes, personal communication; Table **S1**). Structurally, these formations are very similar to microbial communities previously observed in other environments, such as shallow hydrothermal vents [5], are dominated by filamentous bacteria from the genera *Beggiatoa* *,* *Thioploca* *and* *Thiomargarita* ; and microbial communities observed at fjords in Greenland are dominated by filamentous bacteria from the Class *Gammaproteobacteria* [6]. Little is known about the detailed phylogenetic and metabolic diversity of these conspicuous microbial communities in other ecosystems, and no studies have been carried out at the Comau fjord in Chile.

Culture independent approaches, such as metagenomic analysis [7], can help describe the microbial community structure by providing information on the major taxonomic groups and assessing the metabolic diversity present in environmental samples. In this work we take advantage of the power of metagenomics to describe a previously unknown microbial mat community discovered at the Comau fjord.

## Material and Methods

### Sample collection and nutrient measurements

**Figure 2:**
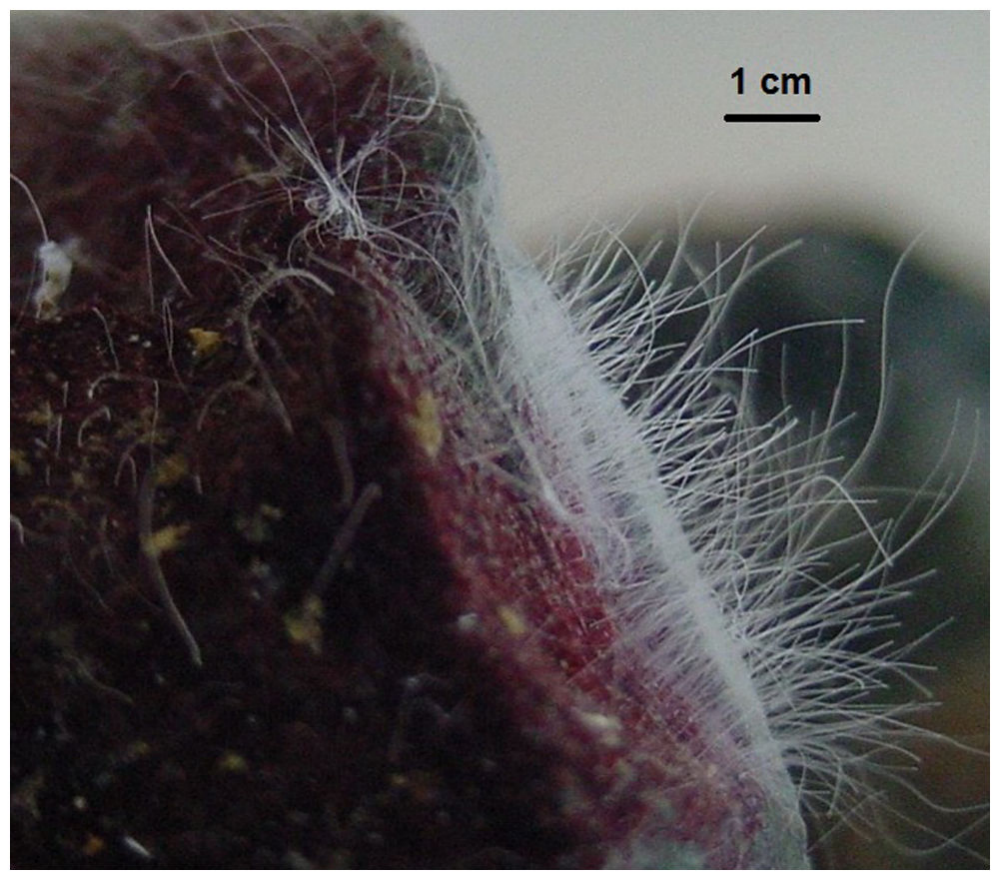
Filamentous sulfur formations observed in the microbial mats. (Photo by V.A. Gallardo).

Samples of filamentous microbial mats were collected in October 2011 by SCUBA, at depths between 25 to 29 m at the Comau Fjord (42^°^19,894´ S, 72°27,661’ W) on board of the R/V *Mytilus*, property of the Huinay Foundation. No specific permits were required for the described field studies and these studied locations are not privately owned. Additionally, the study did not involve endangered or protected species. All samples were kept between 0 and 4 °C while in transit to the laboratories at the Universidad de Concepción, in Concepción, Chile (~3 days). Images of the mats were captured using an Olympus trinocular microscope, under 40X magnification (Figure **2**).

### DNA isolation and sequencing

A single sample from the microbial mat was subdivided in three fragments of approximately 0.5 g each. Each fragment was concentrated by centrifugation and washed three times with PBS/Tween® 20 (0.1%), and genomic DNA was extracted using the FastDNA Spin Kit (MP Biomedicals, Santa Ana, CA.), and quantified with a Infinite F2000pro Tecan reader (Tecan Group Ltd, Switzerland). The obtained DNA was combined to obtain a total of 14 µg and sequenced by Omics Solutions (Santiago, Chile) on a 454 GS-FLX+ platform (454 Life Sciences, Bradford, CT, USA), generating 982,663 reads with an average length of 555.74 nt. Raw reads are available at the NCBI Short Read Archive (SRA) under the accession number SRX243534.

### Assembly and Annotation

Raw metagenomic reads were filtered to remove duplicate and low quality sequences, as well as trimmed to remove low quality positions, using PrinSeq [8] (Parameters: -min_len 60, -max_len 895, -min_qual_mean 20 –ns_max_p 1, -derep 12 –trim_qual_right 25, -trim_qual_rule lt –trim_qual_window 1, -trim_qual_step 1). Sequence assembly was performed using Newbler (Version 2.7, Roche; parameters: min overlap 40, min overlap identity 90%, seed step 6). Assembly statistics are summarized in Table **S2**. Assembled contigs and unassembled reads were annotated using the IMG/MER portal [9], and are available under the accession number 3300000270. The genomes of *Colwellia* *psycherythraea* 34H (IMG Taxon ID: 637000081) and *Moritella marina* MP-1 (IMG Taxon ID: 2519899695) were retrieved from IMG/ER and recruitment of metagenomic reads against these reference genomes was performed using Nucmer 3.0 [10], and visualized using Circos [11]. The number of reads in the metagenome with matches to proteins in the reference genomes, was estimated using a Blastx [12] search of all the metagenomic reads against the proteins of each genome, selecting hits with an e-value of less than 1E-05 and similarity over 80%.

### Phylogenetic analysis and annotation of the reads

To estimate the taxonomic composition of the microbial mat sample, two methods were performed on the unassembled data set. First, 16S rRNA gene sequence fragments were extracted from the complete set of unassembled reads by a Hidden Markov model search (HMM) implemented in the WebMGA server [13]. These fragments were aligned against a reference alignment using NAST [14] and classified using Greengenes (December 2011 version) [15] (≥ 75% similarity over a minimum length of 200 bp). To complement the read-based 16S rRNA gene analysis, the compositional-based method MGTAXA [16], was used on the complete set of unassembled reads. This approach provided a global analysis of the abundance of the different taxonomic groups in the community, including viruses. All metagenomic reads were annotated using the RAMMCAP pipeline, implemented in the CAMERA portal [17]. All annotation and taxonomic assignments generated by RAMMCAP and MGTAXA are available from the Dryad Digital Repository (http://dx.doi.org/10.5061/dryad.pk8qv).

### Metabolic reconstruction and comparative analysis

Metabolic pathway reconstruction was done using MinPath (version 1.2) [18], based on the KO number annotation generated by the IMG/MER annotation. Visualization of metabolic pathways was done using the KEGG server [19].

Under- and over-represented functional categories in the Comau metagenome were evaluated using an odds ratio test [20]. COGs numbers (clusters of orthologous groups) were collected for all available bacterial genomes in the IMG-ER database and were compared with the COG annotation of the Comau community using an odds ratio (*A*/*B*)/(C/D), where *A* is the number of hits in a given COG category for all the bacterial genomes, *B* is the number of hits for all COG categories in all the bacterial genomes, *C* is the number of hits to the same category for the Comau metagenome and *D* is the number of hits to all COG categories in the Comau metagenome. A category was considered over-represented for odds-ratio values over 1 and p-values less than 0.05. All calculations were done using the statistical package R [21] (version 2.15.2).

## Results and Discussion

### Comau microbial mats

Visual observations in the study area showed that the microbial mats can be found at depths between 1–100 m, usually on rock walls with a slope greater than 90^°^. Seepage of water rich in H_2_S of hydrothermal origin was observed close to the location of this microbial mats [2,3], with chemical compositions that could be supporting the metabolism of the microbial community, according to measurements performed in October 2012 (Table **S1**).

The mats showed an ellipsoid shape, with vertical extensions of up to 1 meter and 0.5 meters in width. These microbial mats were firmly anchored to the rocky substrate, forming “cotton-like lumps” stones (Figure **1**), which can be observed without any optical magnification, or long white threads of several mm (Figure **2**), that can reach lengths up to 10 cm. Filaments usually adhered to clean rock surfaces, but in some cases they were also seen anchored to the shells of benthic invertebrates such as chitons and mussels. The morphology of these microbial mats, as well as the source of water rich in H_2_S, shows similarity with previously studied microbial communities in other locations, dominated by sulfur-oxidizing filamentous bacteria [5].

### Metagenomic sequencing

Microbial mats samples were collected and total community DNA extracted and pyrosequenced as described in the Methods section. After quality filtering and trimming, a total of 954,266 sequences were retained, comprising a total of 461 Mbp, with an average read length of 483.97 bp.

Taxonomic composition of the microbial community was estimated using two complementary methods. First, we identified all the reads within the metagenome that contained 16S rRNA gene sequence fragments. These sequences were obtained from unamplified DNA, meaning that potential PCR amplification biases were avoided. Therefore, the abundance and identity of this marker gene subset should be representative of the microbial membership present in the sample [22]. A total of 2,399 16S rRNA gene reads were identified, representing 0.25% of all quality-filtered reads in the sample. This recovery rate similar to what has been observed in other metagenomic studies [23]. After alignment and filtering, a total of 1,869 16S rRNA reads ≥200 nt in length were recovered (Table **S3**).

**Figure 3:**
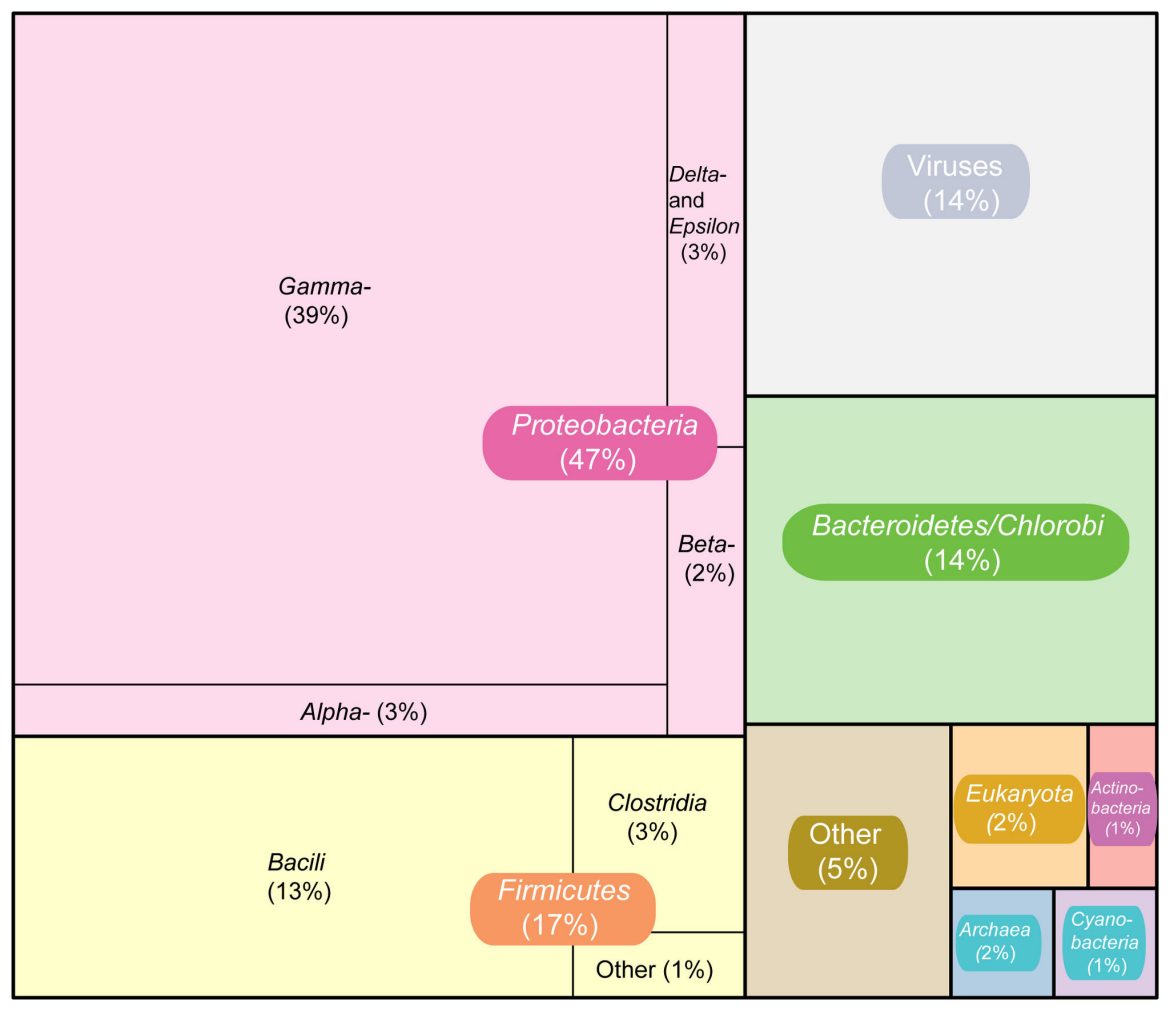
Recruitment of metagenomic reads against the genomes of *C* *. psycherythraea* and *M. marina*. Genome is colored according to COG categories, and blue lines indicate reads from the metagenome that were recruited to the reference genome at ≥ 80% identity.

Complementing the 16S rRNA gene analyses, the complete classification of the reads using MGTAXA showed that the community is dominated by members of the Bacteria (83%), with only 2% of the sequences classified as Eukaryotes and 1% as Archaea (Figure **3**, Table **S4**). These results are aligned with those obtained from the 16S rRNA gene analysis, where only one sequence (out of 1,869) was assigned to the Archaea. Approximately 14% of the reads were classified by MGTAXA as sequences of viral origin. This is similar to observations for other marine microbial communities [24], with nine families that have at least 1% of the reads classified into them (Figure **S1**). Among all the classified viral reads, the most abundant family is Siphoviridae (38.7%), followed by the Myoviridae (14.35%), and the Podoviridae (5.74%). These three families are members of the Order Caudovirales, double-stranded DNA tailed phages highly abundant in marine ecosystems [25]. Among the less abundant groups, we found members of the Poxviridae (1.1%), Herpesviridae (1.1%), and Baculoviridae (1.4%), which are double-stranded DNA viruses described as pathogens for Eukaryotes such as Protists, Molluscs and Crustacea [26]. Among the RNA viruses, we found Reoviridae (7.7%) and Coronaviridae (3.3%), both groups that also affect Eukaryotes [26,27].

For the bacterial classified metagenomic reads, the most abundant group at the Class level was the *Gammaproteobacteria*, with 38.6% of the reads classified into this group. Comparison of the Huinay community to previously characterized microbial communities from sulfide-rich environments [5,6], shows a similar composition at the Class level, however the most abundant organisms were from the Order Thiotrichales, which in the community sampled in our study, only represented less than 1% of the reads (0.96%). This suggests that although these communities can have common morphological characteristics, their taxonomic compositions can be different. Explanations for these differences could probably be found in the chemical composition of the surrounding water. Currently, we need more detailed information about the water composition that surrounds the Comau microbial mats, but this is a target to consider for future sampling endeavors.

Analysis of the 16S rRNA reads at the genus level indicated that the most abundant genera found in the community include *Moritella* (~32%), *Colwellia* (~15%), *Vibrio* (~6%) and *Arcobacter* (~5%). *Moritella* is a diverse genus, with species found associated with deep-sea wood falls [28] and deep sea microbial mats [29]. The two sequenced genomes for this genus, *M. marina* MP-1 (ATCC 15381) [30] and *Moritella* *sp.* PE36, both organisms isolated from deep-sea environments. *Colwellia* is a diverse genus with isolates obtained from various marine systems, including Antarctic environments [31], marine invertebrates [32] and tidal flats [33]. The only sequenced genome for this genus, belongs to *Colwellia* *psycherythraea* 34H, a psychrophilic bacterium isolated from Antarctic sediments [34]. The genus *Arcobacter* has been associated with sulfide-oxidizing microbial mats [35], as well as microbial mats from other fjord ecosystems [6]. Common characteristics among the most abundant genera, *Moritella* and *Colwellia* , include their presence in deep sea and cold environments and their ability to catabolize diverse organic compounds [36]. These properties could explain their presence in the Comau fjord microbial mat community, related to the input of organic material input from the surrounding hills into the waters of the fjord. That the two classification methods differ in their ability to estimate the abundance of different phylogenetic groups may be partially due to differences in the copy number of the 16S rRNA gene in the microorganisms present in the community [37], and may overestimate the abundance of microbial groups that contain more than one copy per genome. A clear example of this can be found for the case of *C. psychrerythraea* 34H, which has six copies of the 16S rRNA gene in its genome. Nevertheless, both approaches agree on the ranking of the top phylogenetic groups that are present in the community (Table **S4**).

### Genome recruitment and metagenome assembly

In light of the results for the phylogenetic classification, we decided to try to reconstruct genomes from the environmental sequences by taking all the metagenomic reads and recruiting them to a reference genome. This was done for the two most abundant representatives of the community, *M. marina* MP-1 [30] and *C* *. psycherythraea* 34H [34]. Recruitment of all the metagenomic reads against these genomes resulted in a partial coverage of both reference genome at 90% identity or higher (Tables **S5** and **S6**). In the case of *C* *. psycherythraea* , a total of 10,572 reads were mapped (1.1% of all the reads), while in the case of *M. marina*, a total of 31,077 reads were mapped (3.2% of all the reads).

**Figure 4:**
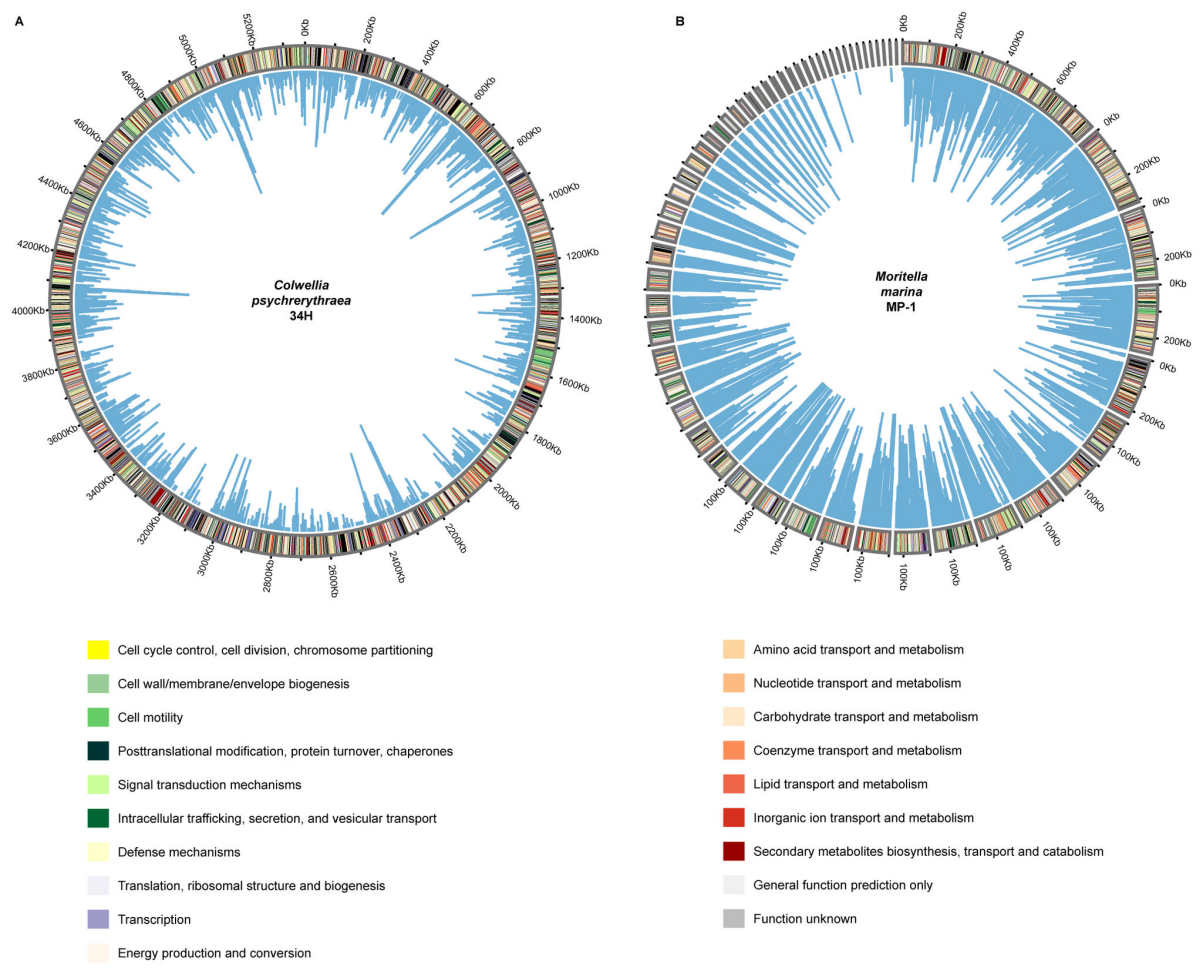
Community composition based on the metagenomic reads classified using MGTAXA **[16]**. Percentage of sequences classified into Phylum and Class are shown next to the corresponding category.

For the *C* *. psycherythraea* genome (Figure **4, A**), the low number of recruited reads (only 1.1% of all the reads), in contrast with the high abundance of this organism based on the 16S rRNA analysis, can be explained by the high copy number of this operon in this organism. Read mapping showed that 1,117 genes (out of 5,066 present in the genome) did not have any matches with sequences from the Comau metagenome (Table **S7**). Most of these genes encode for hypothetical proteins, dispersed throughout the *C* *. psycherythraea* genome, which could be indicative of the putative metagenomic islands; genomic regions that are found in the *C* *. psycherythraea* genome, but absent in environmental populations [38], such as the community sampled in this study. An alternative explanation is that the sampled *Colwellia* population belongs to a different species, which could also explain the differences found in the recruitment analysis. With the available data, is difficult to be more conclusive in this issue, and further exploration is needed, including a better representation of the *Colwellia* population present in the community using microscopy and cultivation approaches. Within the regions where metagenomic reads were absent compared to the genomic reference, we identified genes coding for proteins involved in the synthesis of cell surface components, as well as genes encoding hypothetical proteins and phage-related proteins (such as integrases and helicases). These genes have also been identified previously as located within metagenomic islands in other microorganisms [39,40]. Among some of the other genes that did not recruit any reads from the metagenome, we found genes coding for glycosyl-transferases and proteins involved in the synthesis of polysaccharides, both of which have been suggested to be related to the adaptation to cold temperatures in *C* *. psycherythraea* [34]. This could suggest adaptation of the *Colwellia* populations present in the sampled community to different temperature regimes as the average water temperature for these communities (12-18^°^C) is higher than the optimum growth temperature for *C* *. psycherythraea* (~8^°^ C) [34]. An interesting genomic region that is present in *C* *. psycherythraea* but did not recruit any metagenomic reads, contains the genes coding for the TorECAD proteins, which take part in the respiration of trimethylamine n-oxide (TMAO) [41]. This suggests that the *Colwellia* population found in these microbial mats may lack the ability to utilize TMAO as an alternative electron acceptor under anaerobic conditions. The absence of this gene cluster was confirmed by blast-based analysis of the reference *tor* genes against the complete metagenomic data set. Measurements performed in the surrounding waters of the microbial mats, suggest that oxygen is always present (Javier Sellanes, personal communication), but long-term measurements are needed to evaluate possible seasonal variations in oxygen availability.

Recruitment of reads to the *Moritella marina* MP-1 (Figure **4, B**) genome showed that 433 genes (out of 4,245 present in the genome) did not have any matches with the metagenome (Table **S8**). This suggest a very similar situation to the *C* *. psycherythraea* read recruitment, with the presence of metagenomic islands that are present in the reference genome of *M. marina*, but are not present in environmental populations [38]. Among the absent genes in the environmental population, we found genes encoding hypothetical proteins and phage-related proteins, as well as genes coding for proteins involved in cell-wall biosynthesis, features described to be present in these metagenomic islands [38,39]. Among the mapped features of the *M. marina* genome, we found that the complete set of *pfa* genes, which encode for proteins that take part in the production of the omega-3 polyunsaturated fatty acid docosahexaenoic acid (DHA; 22:6n-3) [42] were presented in the metagenome sequence based on the read recruitment. *M. marina* MP-1 has been described as a producer of high amounts of DHA [30,43], which could contribute to its membrane homeostasis under low temperature and high pressure conditions. The mapping of reads to these genes, suggest the ability of the *Moritella* community present in the microbial mat to produce DHA, although its functional role within the community needs to be further explored.

A complementary approach to read recruitment is to assemble the full metagenome with the goal of recovering not only single genes, but also operons. This approach could shed some more light on the metabolic potential of the community, as could be the case for DHA production. Assembly of the metagenomic sequences resulted in a total of 19,431 contigs (Table **S2**), representing 23.1% of the reads and 22.1% of the total bases in the metagenome data set. The N50 for the assembly was 1,529, and the majority of the assembled and annotated contigs contain a single gene (Figure **S2**). For the operon encoding proteins involved in the synthesis of DHA, no contigs representing the complete operon were found.

Contig annotation, particularly the largest ones, shows the presence of several genes encoding viral proteins. This result is also supported by the classification of the metagenomic reads, which shows that 14% of the reads where of viral origin. To further look for evidence of contigs of viral origin, we searched for viral proteins in the assembled contigs, against the 1,184 phage genomes present in the Phantome database [44]. Of the 40,200 predicted proteins in the assembled contigs, 8,898 had hits against phage proteins in the Phantome dataset. Several of the proteins predicted in the contigs, had matches with the phage database (Table **S9**). As an example of these matches, for the largest contig in the assembly (HuiMet_100001; 39,478 bp; 71 genes), 35 of its proteins had matches against the viral dataset, and in particular 14 of these hits (average identity 41.1%) were against the *Vibrio* phage VP58.5, a lysogenic phage that infects *V. parahaemolyticus* O3:K6 strains recovered in Chile [45]. Similarly, in the case of the second largest contig (HuiMet_100002; 25,997 bp; 33 genes), 21 of its proteins had matches against the viral proteins, with 14 of them versus *Vibrio* phages (Table **S9**).

The overall distribution of these viral hits suggests the presence of several viral species in the sample, including *Bacillus* , *Campylobacter* and *Vibrio* phages (Table **S10**). Some of these proteins hits could be representative of integrated phage elements, and not true viruses that are present in the community. To answer the question of how abundant are viruses in these microbial mats, a more targeted approach to study the viral diversity present in the community will be needed.

### Metabolic analysis of the Comau microbial mat community

At the COG level, we can look at the total number of sequences assigned to functional categories in the complete metagenome, including both assembled contigs (corrected by abundance) and unassembled reads as shown in Table **1**. The most abundant functional categories in the community are amino acid transport and metabolism (9.6%) and energy production and conversion (7.5%). Additionally, a large percentage of sequences were assigned to the general function prediction (10.1%) and unknown function (5.8%) categories.

**Table 1:** COG annotation obtained from IMG, of the assembled and unassembled sequences in the Comau metagenome.

	**COG category**	**Assembled**	**Unassembled**	**Total**
Cellular processes and signaling	Cell cycle control, cell division, chromosome partitioning	1,328 (1.2%)	4,473 (1.1%)	5,801 (1.1%)
	Cell wall/membrane/envelope biogenesis	6,572 (5.7%)	29,099 (6.6%)	35,671 (6.4%)
	Cell motility	2,500 (2.2%)	8,801 (2.1%)	11,301 (2.0%)
	Posttranslational modification, protein turnover, chaperones	4,866 (4.2%)	18,361 (4.2%)	23,227 (4.2%)
	Signal transduction mechanisms	6,374 (5.6%)	28,972 (6.5%)	35,346 (6.3%)
	Intracellular trafficking, secretion, and vesicular transport	3,305 (2.9%)	11,132 (2.5%)	13,437 (2.6%)
	Defense mechanisms	2,084 (1.8%)	10,903 (2.5%)	12,987 (2.3%)
Information storage and processing	Translation, ribosomal structure and biogenesis	9,555 (8.3%)	27,672 (6.2%)	37,227 (6.7%)
	Transcription	7,299 (6.4%)	24,411 (5.5%)	31,710 (6.7%)
Metabolism	Energy production and conversion	8,776 (7.7%)	33,629 (7.6%)	42,405 (7.6%)
	Amino acid transport and metabolism	10,397 (9.1%)	43,021 (9.7%)	53,418 (9.6%)
	Nucleotide transport and metabolism	4,014 (3.5%)	12,566 (2.8%)	16,580 (2.9%)
	Carbohydrate transport and metabolism	4,651 (4.1%)	21,708 (4.9%)	26,359 (4.7%)
	Coenzyme transport and metabolism	5,418 (4.7%)	21,708 (4.9%)	27,126 (4.9%)
	Lipid transport and metabolism	4,084 (3.6%)	16,805 (3.8%)	20,889 (3.8%)
	Inorganic ion transport and metabolism	5,026 (4.4%)	22,467 (5.1%)	27,493 (4.9%)
	Secondary metabolites biosynthesis, transport and catabolism	1,870 (1.6%)	8,918 (2.1%)	10,788 (1.9%)
Poorly characterized	General function prediction only	11,038 (9.6%)	45,235 (10.2%)	56,273 (10.1%)
	Function unknown	7,127 (6.2%)	25,246 (5.7%)	32,373 (5.8%)

**Figure 5:**
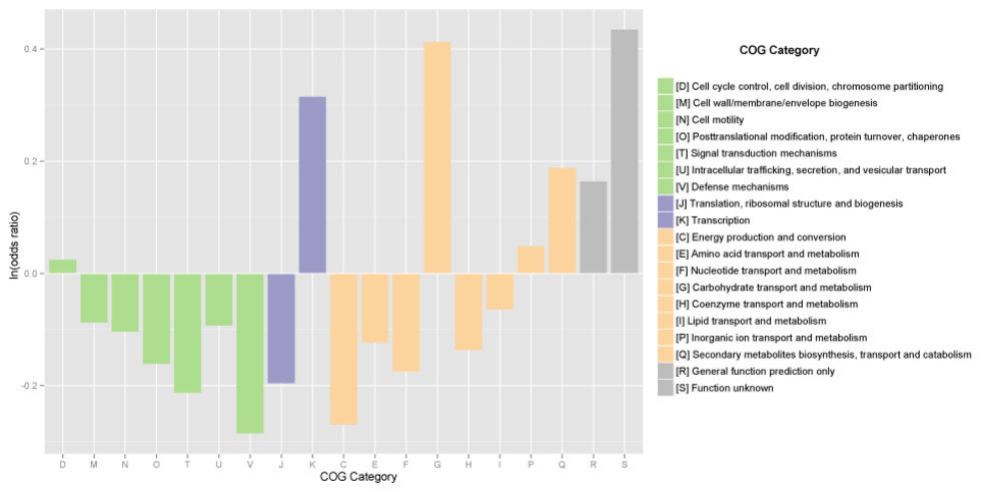
Odds ratio of COG categories of all sequenced bacterial genomes versus the Comau metagenome. Values over 0 indicate categories that are enriched in the bacterial genomes versus the Comau metagenome, and values less than 0 indicate categories where the Comau metagenome is enriched versus all the bacterial genomes. With the exception of the cell cycle category, all other groups showed significant changes by two-tailed Fisher exact test, at 95% confidence level.

Based on COG categories we compared the abundance profile of each functional category in the metagenomic sample, with all the bacterial genomes available in IMG-ER [20]. The results showed that several categories were enriched in the Comau community (Figure **5**), including cellular processes (such as cell wall/membrane/envelope biogenesis and defense mechanisms) and metabolic functions (such as energy production and amino acid transport and metabolism). Interestingly, a comparison of the Comau metagenome COG classifications with 21 other metagenomic data sets revealed the greatest similarity with whale fall ecosystems (Figure **S3**). The similarity between these two communities is at present difficult to explain except for the common presence of reduced sulfur compounds and the potentially rich sources of organic compounds from the cold-temperate rain forest ecosystem that can be found around the Comau fjord.

A complete overview of the metabolic potential of the community can be generated using the functional annotation of its predicted proteins. Based on the IMG annotation of KO numbers, we reconstructed the metabolic pathways that are present in the community using MinPath [18]. The overall reconstruction at the community level, shows the presence of complete glycolysis, TCA cycle and pentose phosphate pathways (Table **S10**), among other carbohydrate metabolism pathways. Carbon fixation was found to be present via the reductive carboxylate cycle and evidence of methane metabolism was also found in the metabolic reconstruction. Pathways for degradation of organic compounds such as Bisphenol, Fluorobenzoate, and other organic contaminants were found to be present in the community as well. Currently no data is available on the occurrence of these compounds in the region, but the presence of these pathways may suggest either current or past encounters with these chemicals. Further measurements of organic compounds in the water column and possible *in-situ* experiments will be needed to explore this.

**Figure 6:**
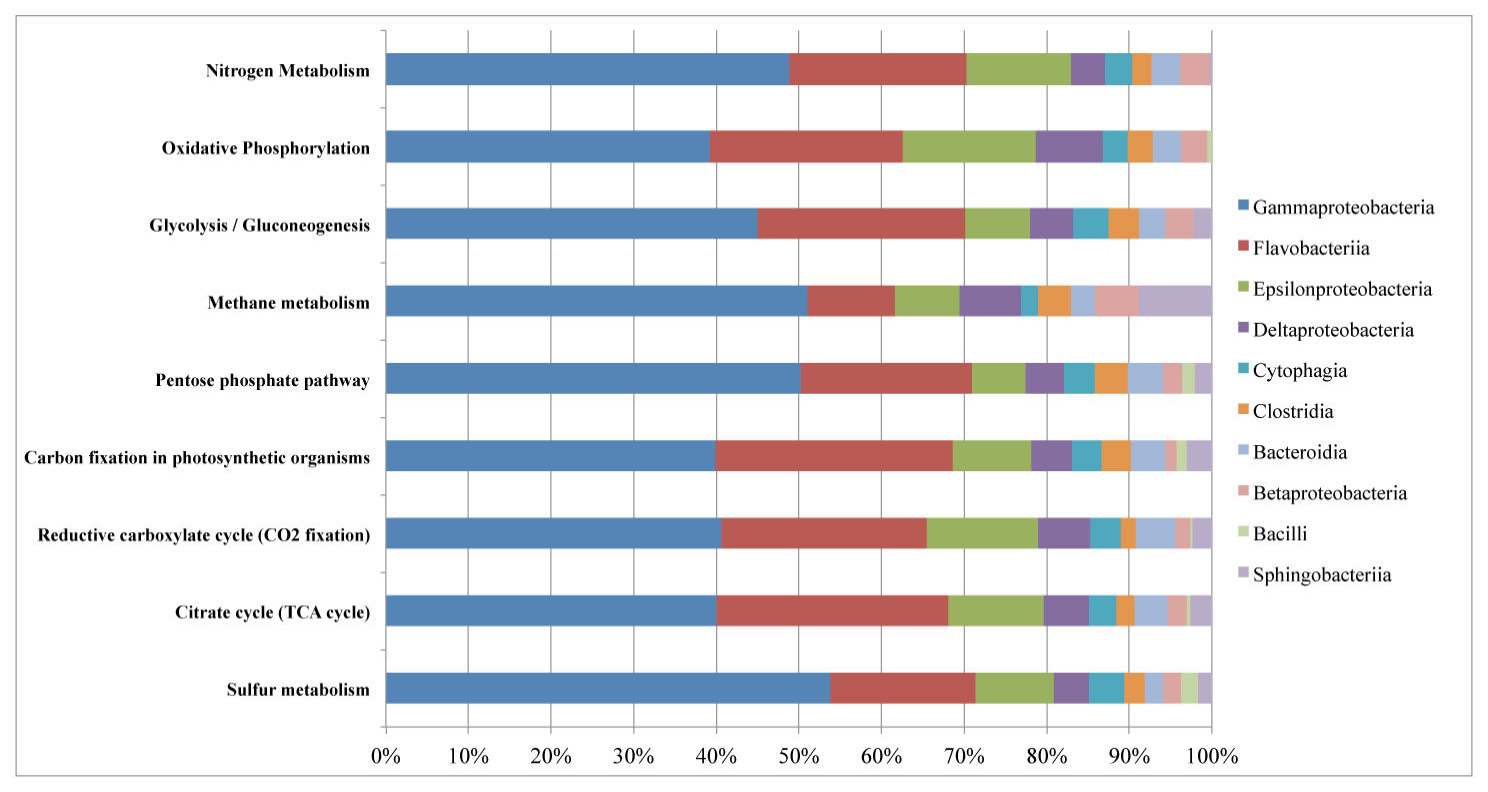
Taxonomic classification of KEGG pathways related to energy metabolism. Each pathway was determined to be present in the community based on the MinPath analysis [18]. Taxonomic affiliation of each sequence was based on the results provided by the IMG-MER annotation [9].

To further understand the interactions between the Comau microbial community and the surrounding environment, we looked in detail at some of the pathways involved in carbon, nitrogen and sulfur metabolism. By combining the taxonomic annotation and the functional annotation of all the metagenomic reads, we evaluated the metabolic potential of the community for these functional processes. The dominant groups associated with these pathways where *Gammaproteobacteria*, followed by the *Flavobacteria* (Figure **6**, Table **S11**). For example, analysis of the carbon fixation pathways, suggests that the community is capable of acquiring carbon via CO_2_ fixation, in particular using the reverse TCA pathway, as only a few hits were detected for the RuBisCO complex (Figures **S4** and **S5**).

Nitrogen is a key nutrient in microbial communities, and its cycling is driven by multiple transformations that are carried out by microorganisms, including fixation, denitrification and assimilation [46]. A critical component of this cycle is the partitioning of inorganic di-nitrogen into a bioavailable form, a processed mediated by the nitrogenase enzyme complex involved in nitrogen fixation. This complex is encoded by the *nif* operon, and its genes can be used as markers to identify potential nitrogen fixation in a microbial community [47]. Based on the KO annotation, we did not find any members of the nitrogenase complex, suggesting the lack of capability for nitrogen fixation in this microbial community, and that most of the nitrogen utilized by members of this community is derived from nitrate, nitrite and ammonia (Figure **S6**). We looked at the potential of the community to use nitrogen compounds as a source of energy, where the potential for dissimilatory nitrate reduction (NapAB complex) is present, as well as the nitrate reduction to ammonium (NfrA) (Figure **S6**).

We also looked at the distribution and abundance of genes involved in sulfur cycling dynamics. Dissimilatory sulfur-based energy conversion is a process that occurs almost exclusively among Bacteria and Archaea [48], where this metabolism is linked to energy transformation via photosynthesis or respiratory processes. Several mechanisms have been described for the conversion of various reduced inorganic sulfur compounds [49]. Reconstruction of the metabolic pathway for sulfur reactions (Figure **S7**) shows the potential of the community for carrying out the reduction of sulfur compounds, where the dominant members of the community associated with this processes are the *Gammaproteobacteria* and *Flavobacteria* (Figure **6**). No proteins involved in sulfur oxidation reactions, such as the *Sox* system [49], were found in the metagenome, suggesting that the microbial community is not carrying out these set of reactions.

The current data suggest that this community uses two main sources of energy: one from nitrogen compounds and the other through the reduction of sulfur compounds. Further exploration is needed to elucidate the details of these processes, including *in situ* measurements for the presence of sulfur compounds, complemented with culture-independent approaches. Also, a more careful analysis of the microbial mats will be needed, because different layers of the mat may have different phylogenetic and functional profiles, information that was missed with the approach used in the current study.

In the current study we do not have the resolution to separate.

### Concluding Remarks and Future Perspectives

In the present study, the taxonomic diversity and metabolic potential of the microbial mat community discovered in the Comau fjord was studied using culture-independent methods. This is an area of research that deserves more attention in the region, and we expect that the data and results generated from the current analysis will provide a foundation for future studies. Further characterization of the microbial community, and also more detailed investigations of the geochemical fluxes and allochthonous nutrient inputs that impact the Comau fjord ecosystem needs to be performed to better understand how these spectacular mat communities interact with the aquatic environment. Cultivation approaches will provide a better understanding of the diversity and metabolic potential of some of the dominant members of this community, such as *Colwellia* and *Moritella* , and will help to better define their role in their community, perhaps revealing biotechnological potential [34,50,51]. Seasonal variations in the Comau fjord may also influence the microbial component of the community, changing its taxonomic composition and metabolic potential. Extended temporal studies will be required to provide a more detailed picture of the community and its seasonal dynamics. Finally, a comprehensive view of this ecosystem, will benefit from expanding these studies to include additional community members—ranging from unicellular eukaryotes (such as protozoan grazers) to multicellular organisms such as crustaceans and fishes [52].

## Supporting Information

Figure S1 **Classification of metagenomic reads into viral families.**Reads were classified into viral families using MG-TAXA [16].
(PDF)

Figure S2 **Count of predicted open reading frames (ORFs) in the assembled contigs.** Gene prediction was done using the IMG-MER platform [9].
(PDF)

Figure S3 **Hierarchical clustering of the Comau microbial mat metagenome with related datasets.** All the metagenomes were selected from the IMG-MER website [9], and the hierarchical clustering was done using the tools available on the website.
(PDF)

Figure S4 **KEGG pathway for carbon fixation in photosynthetic organism.** The pathway was generated using the KEGG website [19], and the color intensity reflects the number of proteins associated with a particular enzymatic activity.
(PNG)

Figure S5 **KEGG pathway for carbon fixation pathways in prokaryotes.** The pathway was generated using the KEGG website [19], and the color intensity reflects the number of proteins associated with a particular enzymatic activity.
(PNG)

Figure S6 **KEGG pathway for nitrogen metabolism.** The pathway was generated using the KEGG website [19], and the color intensity reflects the number of proteins associated with a particular enzymatic activity.
(PNG)

Figure S7 **KEGG pathway for sulfur metabolism: reduction and fixation.** The pathway was generated using the KEGG website [19], and the color intensity reflects the number of proteins associated with a particular enzymatic activity.
(PNG)

Table S1 **Nutrient analysis from water samples, October 2012.**
(PDF)

Table S2 **Assembly statistics.**
(PDF)

Table S3 **Classification of 16S rRNA reads with Greengenes.**
(PDF)

Table S4 **Classification of all the metagenomic reads using MGTAXA.**
(PDF)

Table S5 **List of reads mapped to the *C* *. psycherythraea* genome.**
(TXT)

Table S6 **List of reads mapped to the *M. marina* genome.**
(TXT)

Table S7 **List of genes in the *C* *. psycherythraea* genome that had coverage of the metagenomic reads.**
(TXT)

Table S8 **List of genes in the *M. marina* genome that had coverage of the metagenomic reads.**
(TXT)

Table S9 **Assembled and unassembled proteins that had hits against the Phantome viral database [44].**
(TXT)

Table S10 **Count of the viral genomes in the Phantome database [44], with hits to the Comau microbial mat metagenome, indicating the total number of hits and the average sequence identity.**
(TXT)

Table S11 **Taxonomic classification of all the proteins in the KEGG pathways that were predicted to be present in the Comau microbial mat metagenome.**
(TXT)
