# Supplementary figures and images for: Microbial Life in a Fjord: Metagenomic Analysis of a Microbial Mat in Chilean Patagonia
Source: PLoS One. 2013 Aug 28;8(8):e71952. doi: 10.1371/journal.pone.0071952 (PMC3756073; doi:10.1371/journal.pone.0071952)

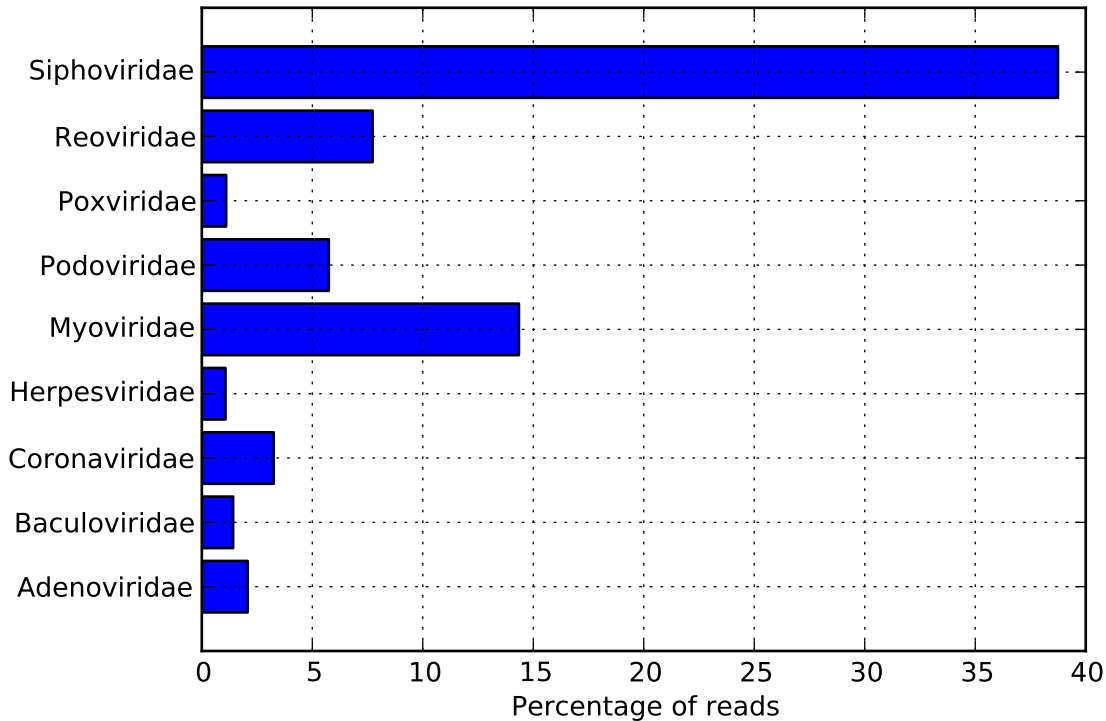

Supplement: Figure S1 — Classification of metagenomic reads into viral families.Reads were classified into viral families using MG-TAXA [16]. (PDF) [file pone.0071952.s001.pdf]

Number of genes in contigs

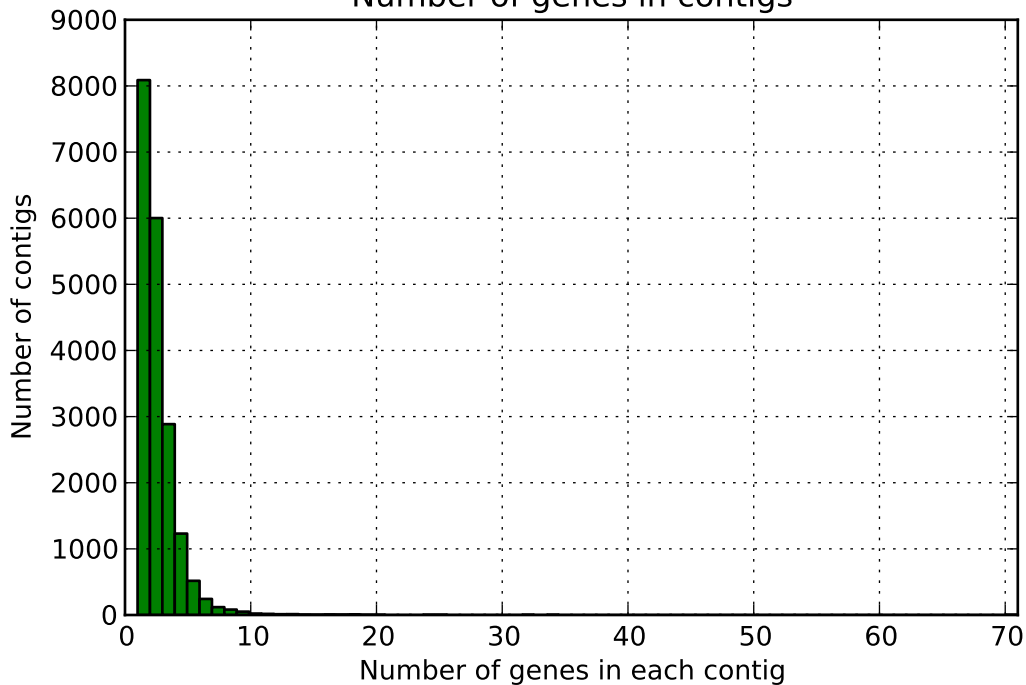

Supplement: Figure S2 — Count of predicted open reading frames (ORFs) in the assembled contigs. Gene prediction was done using the IMG-MER platform [9]. (PDF) [file pone.0071952.s002.pdf]

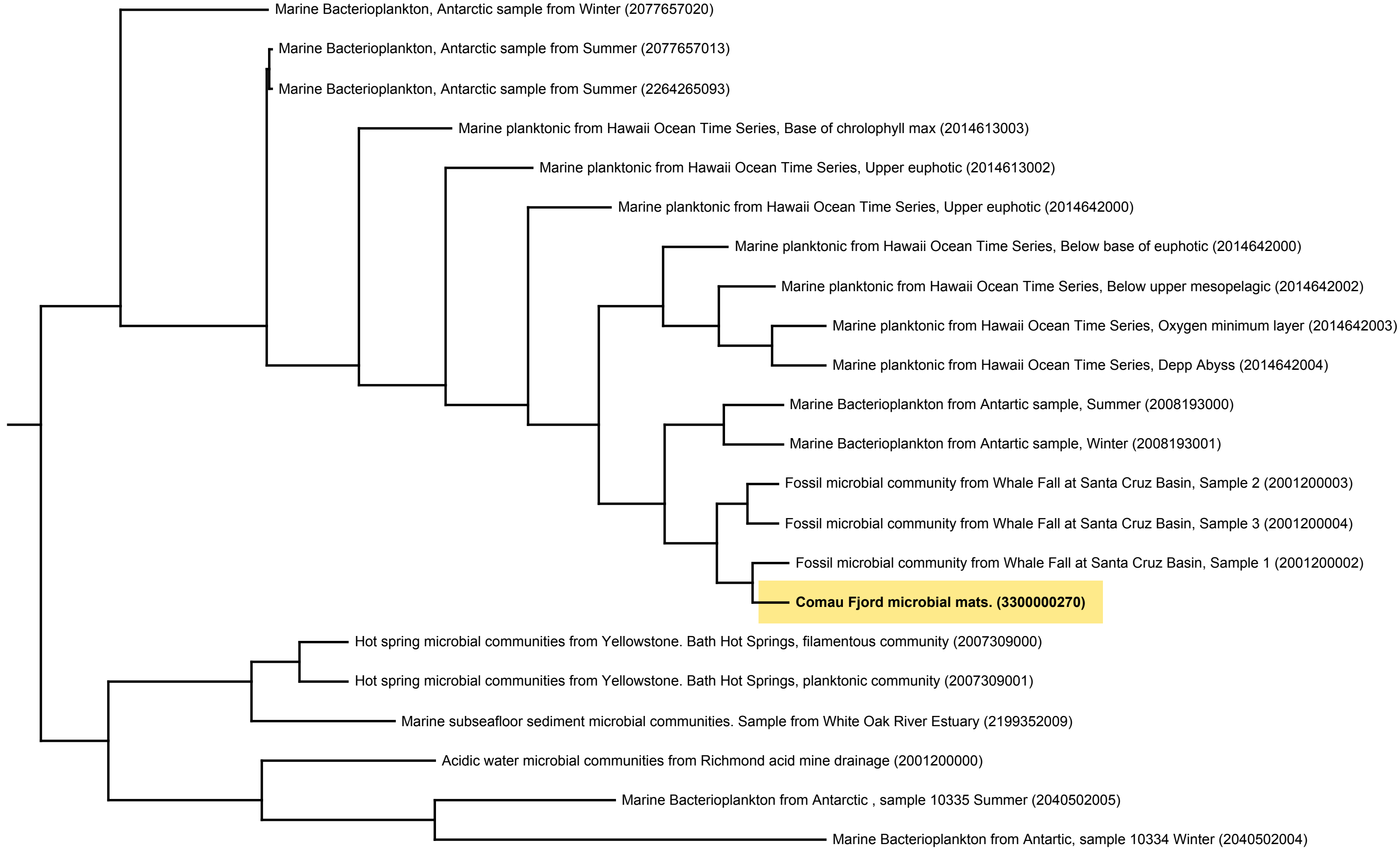

Supplement: Figure S3 — Hierarchical clustering of the Comau microbial mat metagenome with related datasets. All the metagenomes were selected from the IMG-MER website [9], and the hierarchical clustering was done using the tools available on the website. (PDF) [file pone.0071952.s003.pdf]

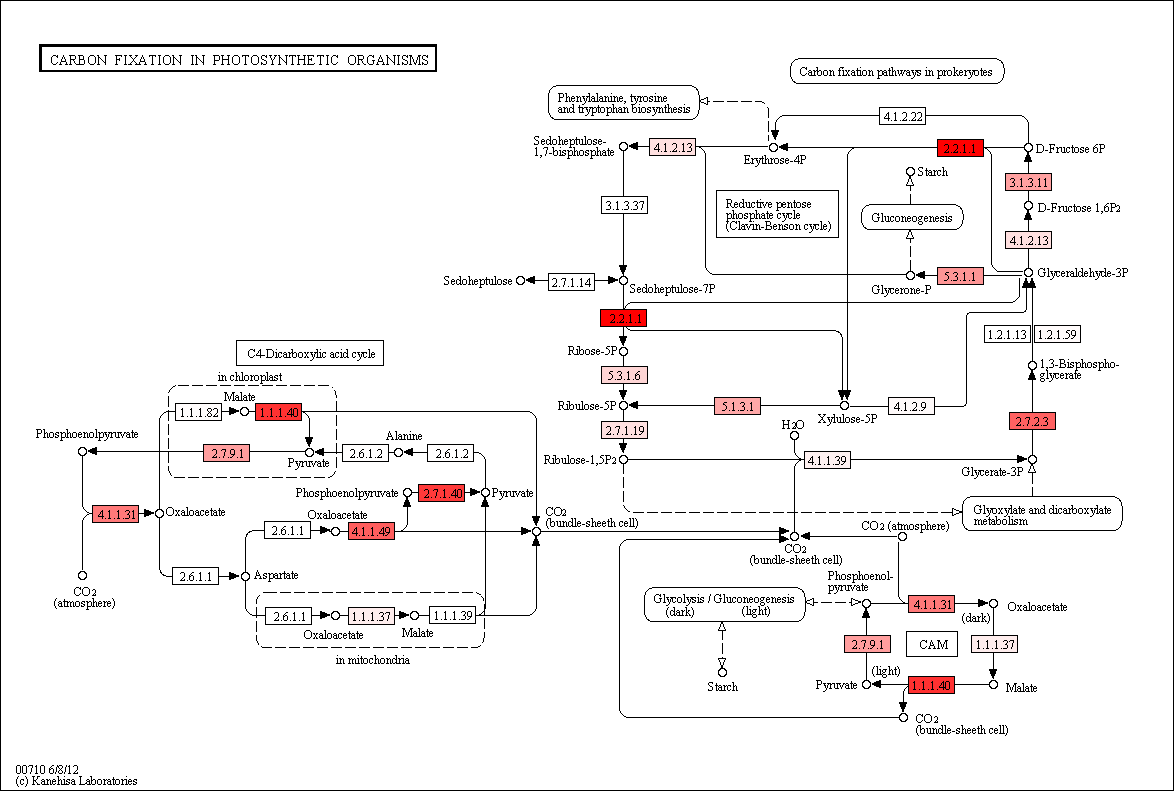

Supplement: Figure S4 — KEGG pathway for carbon fixation in photosynthetic organism. The pathway was generated using the KEGG website [19], and the color intensity reflects the number of proteins associated with a particular enzymatic activity. (PNG) [file pone.0071952.s004.png]

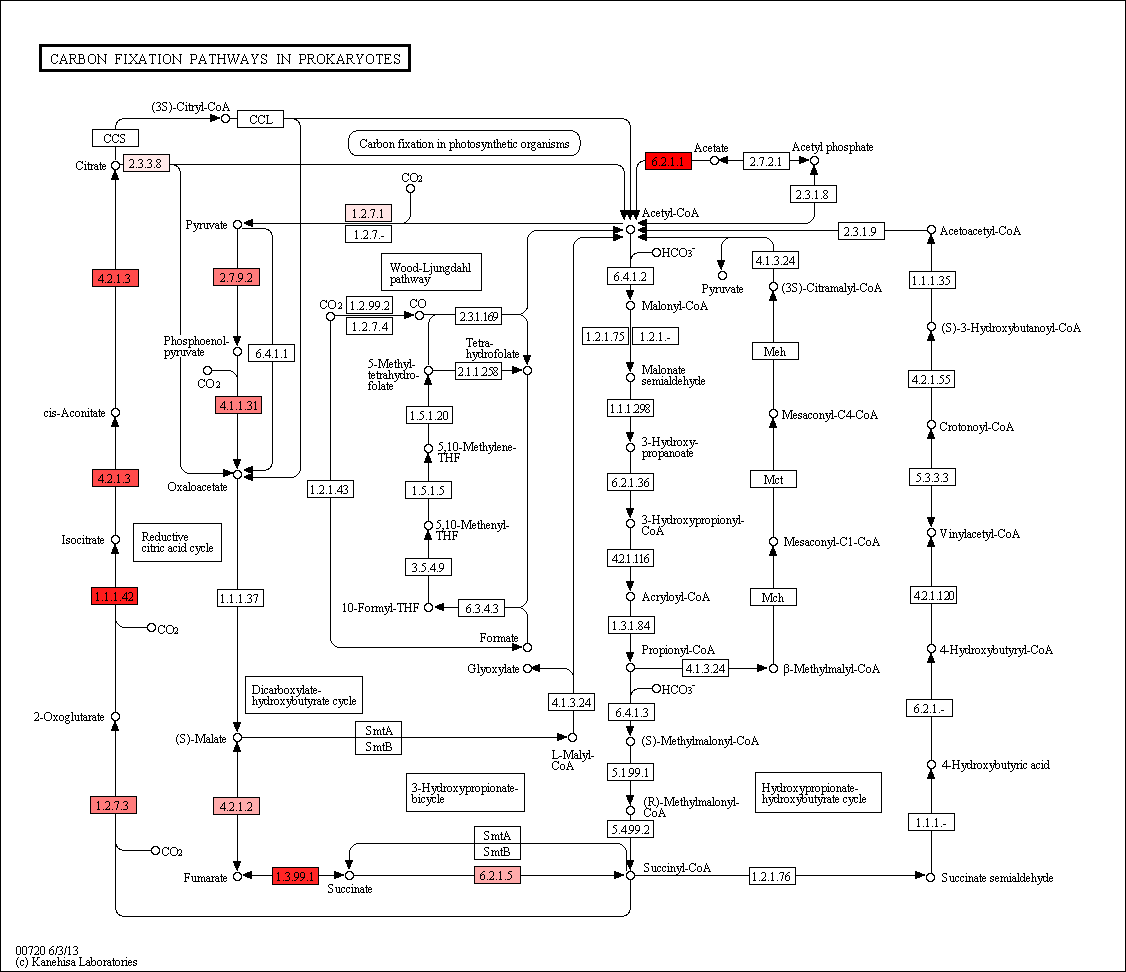

Supplement: Figure S5 — KEGG pathway for carbon fixation pathways in prokaryotes. The pathway was generated using the KEGG website [19], and the color intensity reflects the number of proteins associated with a particular enzymatic activity. (PNG) [file pone.0071952.s005.png]

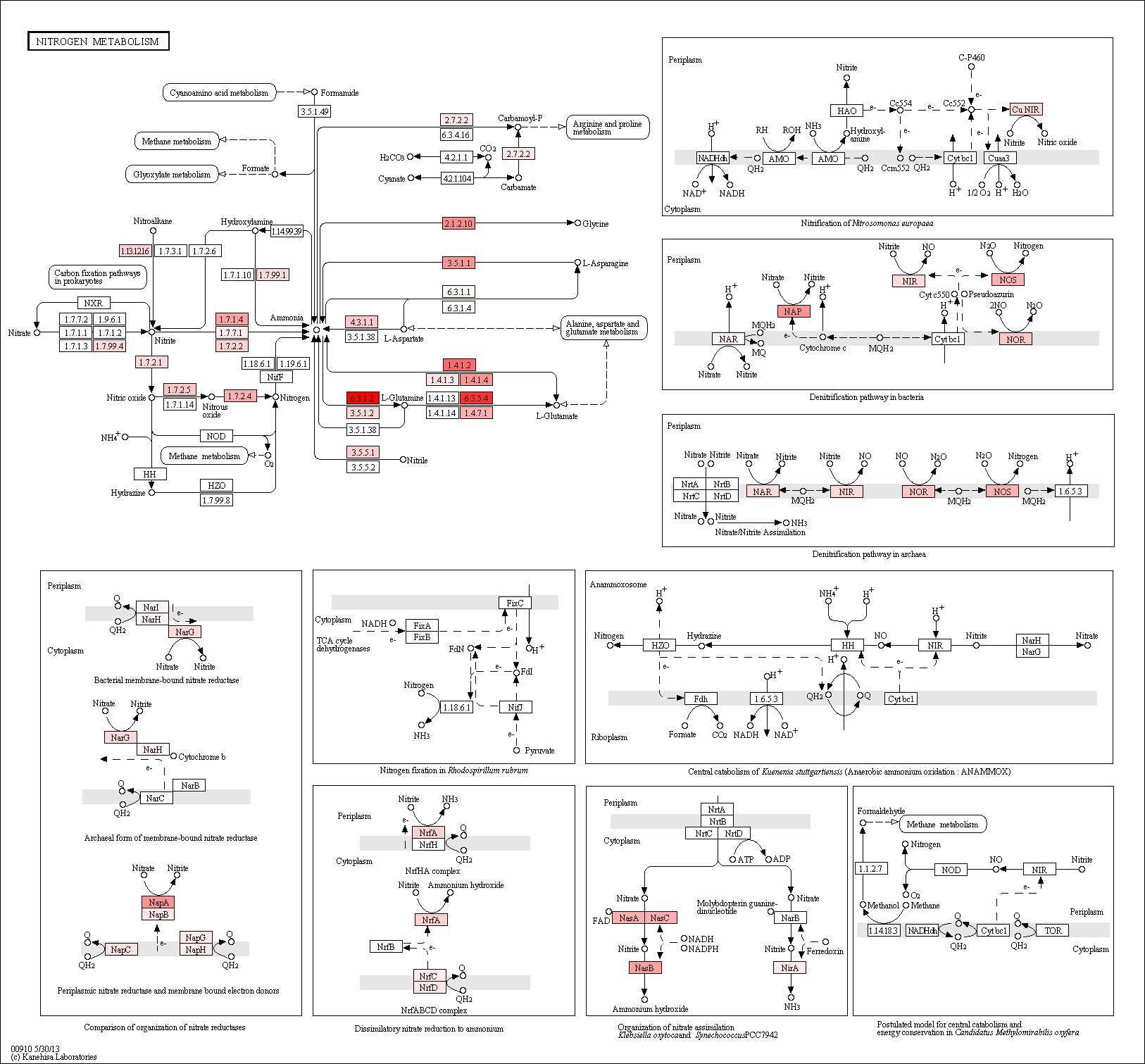

Supplement: Figure S6 — KEGG pathway for nitrogen metabolism. The pathway was generated using the KEGG website [19], and the color intensity reflects the number of proteins associated with a particular enzymatic activity. (PNG) [file pone.0071952.s006.png]

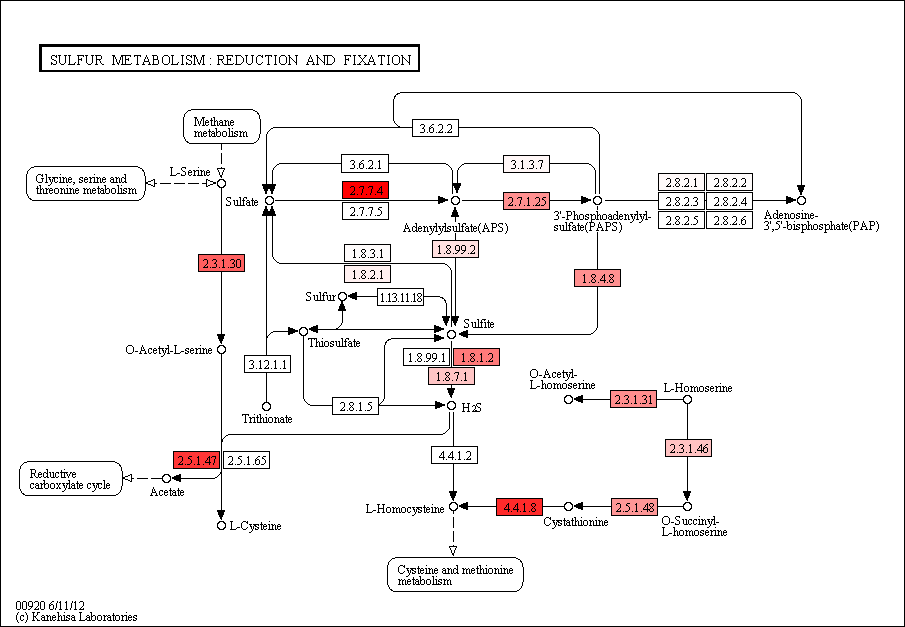

Supplement: Figure S7 — KEGG pathway for sulfur metabolism: reduction and fixation. The pathway was generated using the KEGG website [19], and the color intensity reflects the number of proteins associated with a particular enzymatic activity. (PNG) [file pone.0071952.s007.png]
