# Supplementary material for: Microbial Life in a Fjord: Metagenomic Analysis of a Microbial Mat in Chilean Patagonia
Source: PLoS One. 2013 Aug 28;8(8):e71952. doi: 10.1371/journal.pone.0071952 (PMC3756073; doi:10.1371/journal.pone.0071952)
Supplement: Table S1 — Nutrient analysis from water samples, October 2012. (PDF) [file pone.0071952.s008.pdf]

Supplementary Table 1. Nutrient analysis from water samples, October 2012.

NO_3	8.58 µM
NO_2	0.24 µM
PO_4	1.02 µM
NH_4	2.40 µM
SiO_2	17.67 µM
pH	8.49
$\sum H_2S$	116.11 µM
CH_4	0.26
