# Supplementary material for: Microbial Life in a Fjord: Metagenomic Analysis of a Microbial Mat in Chilean Patagonia
Source: PLoS One. 2013 Aug 28;8(8):e71952. doi: 10.1371/journal.pone.0071952 (PMC3756073; doi:10.1371/journal.pone.0071952)
Supplement: Table S2 — Assembly statistics. (PDF) [file pone.0071952.s009.pdf]

Supplementary Table 2. Assembly statistics

**Aligned bases**	101,071,017
**Number of contigs**	13,513
**Number of bases in contigs**	20,099,859
**Average contig size**	1,487
**N50 Contigs**	1,654
**Largest contig**	39,478
**Total contigs**	19,431
**Singletons**	689,455
