# Supplementary material for: Microbial Life in a Fjord: Metagenomic Analysis of a Microbial Mat in Chilean Patagonia
Source: PLoS One. 2013 Aug 28;8(8):e71952. doi: 10.1371/journal.pone.0071952 (PMC3756073; doi:10.1371/journal.pone.0071952)
Supplement: Table S3 — Classification of 16S rRNA reads with Greengenes. (PDF) [file pone.0071952.s010.pdf]

Supplementary Table 3. Classification of 16S rRNA reads with Greengenes

**Kingdom**	**Phylum**	**Class**	**16S count**
Bacteria	*Actinobacteria*	*Actinobacteria*	2
	Bacteroidetes	*Caldithrixae*	1
		Flavobacteria	288
		Sphingobacteria	16
		Bacteroidia	87
	Chloroflexi	*Anaerolineae*	3
	Firmicutes	*Mollicutes*	1
		Erysipelotrichi	3
		Clostridia	9
		Bacilli	4
	Lentisphaerae	*Lentisphaerae*	3
	Planctomycetes	*Phycisphaerae*	1
	Proteobacteria	*Alphaproteobacteria*	19
		Betaproteobacteria	1
		Deltaproteobacteria	35
		Gammaproteobacteria	1221
		Epsilonproteobacteria	162
	Spirochaetae	*Spirochaetes*	1
Archaea	*Thaumarchaeota*	*Thaumarchaeota*	1
	Verrucomicrobia	*Verrucomicrobiae*	1
		Spartobacteria	1
Unclassified			9
