# Supplementary material for: Microbial Life in a Fjord: Metagenomic Analysis of a Microbial Mat in Chilean Patagonia
Source: PLoS One. 2013 Aug 28;8(8):e71952. doi: 10.1371/journal.pone.0071952 (PMC3756073; doi:10.1371/journal.pone.0071952)
Supplement: Table S4 — Classification of all the metagenomic reads using MGTAXA. (PDF) [file pone.0071952.s011.pdf]

Supp. Table 4. Classification of all the metagenomic reads using MGTAXA

Kingdom	Phylum	Class	Percentage of reads
Bacteria	*Actinobacteria*	*Actinobacteria*	1
	Bacteroidetes	*Bacteroidia*	1
	Cyanobacteria		1
	Firmicutes	*Bacili*	13
		Clostridia	3
		Other	1
	Proteobacteria	*Alphaproteobacteria*	3
		Betaproteobacteria	2
		Delta/epsilon	3
		Gammaproteobacteria	39
	Other		5
Archaea			1
Eukaryote			2
Viruses			14
